# Population structure and fecundity of the Xanthid crab *Leptodius exaratus* (H. Milne Edwards, 1834) on the rocky shore of Gujarat state, India

**DOI:** 10.7717/peerj.16916

**Published:** 2024-02-13

**Authors:** Krupal Patel, Heris Patel, Swapnil Gosavi, Kauresh Vachhrajani, Jigneshkumar Trivedi

**Affiliations:** 1 Department of Life Sciences, Hemchandracharya North Gujarat University, Patan, Gujarat, India; 2 Department of Zoology, The Maharaja Sayajirao University of Baroda, Vadodara, Gujarat, India

**Keywords:** Population structure, Saurashtra coast, Sex ratio, Xanthidae, Breeding, Rocky shore

## Abstract

**Background:**

The population structure and breeding biology of the Xanthid crab, *Leptodius exaratus* (H. Milne Edwards, 1834), on the rocky intertidal region of Shivrajpur in Saurashtra coast, Gujarat state, were examined.

**Method:**

From March 2021 to February 2022, monthly sampling was conducted during low tide using catch per unit effort in the 500 m^2^ area. The sampled specimens were categorised into male, non-ovigerous female or ovigerous female. In order to estimate fecundity, the morphology of the crab specimens (carapace width and body weight) as well as the size of eggs, number of eggs and weight of egg mass were recorded.

**Results:**

A total of 1,215 individuals were sampled of which 558 individuals were males and 657 individuals were females. The size (carapace width) of males ranges from 5.15 to 29.98 mm, while females ranges from 5.26 to 28.63 mm which shows that the average size of male and female individuals did not differ significantly. The overall as well as monthly sex ratio was skewed towards males with a bimodal distribution while unimodal in females. The population breeds year-round, which was indicated by the occurrence of ovigerous females throughout the year. However, the maximum percentage occurrence of ovigerous females was observed from December to April which indicates the peak breeding season. The size of eggs, number of eggs and weight of egg mass were shown to positively correlate with the morphology of ovigerous females (carapace width and wet weight).

## Introduction

Investigation on the population structure of intertidal crabs started in early 1940s (Flores & Paula, 2002), which can reveal the patterns of species interactions and their roles within ecosystems. Accounts on population structure and breeding biology majorly try to understand the genetic diversity, age, spatial distribution, abundance, sex ratio, variation in year-round composition, fecundity of the species, as well as juvenile recruitment (Litulo, 2005; Saher & Qureshi, 2010; Hu et al., 2015; Manzoor et al., 2016). A species’ life history might differ by habitat or even by location. For example, a slight variation in the latitude leading to climatic variation can cause differences between the populations. Variations in the population trends are also due to the effects of several biotic and abiotic factors affecting populations differently (Lycett et al., 2020). Studies on the population structure and breeding biology of a species can help determine its ecological stability in a given habitat and also contribute to our understanding of the species’ biology (Santos, Negreiros-Fransozo & Padovani, 1995; Takween & Qureshi, 2005). This knowledge helps ecologists understand how different species coexist, compete, and interact, influencing ecosystem dynamics. However, such studies have not been carried out so far on some of the commonly occurring brachyuran crabs of Gujarat state.

The Saurashtra coast of Gujarat state, India, is characterised by its rocky intertidal coasts, which support a great diversity of marine organisms, including intertidal crustaceans, especially crab population. With its major inhabiting marine intertidal species, majority of the crab studies have focused on the diversity (Trivedi & Vachhrajani, 2013a, 2013b, 2015, 2016a, 2018; Trivedi et al., 2015, 2020, 2021; Gosavi et al., 2017a, 2017b, 2021; Patel, Patel & Trivedi, 2020, 2021a; Bhat & Trivedi, 2021; Padate et al., 2022). Very less is known about the population structure of these important intertidal organisms (Trivedi & Vachhrajani, 2016b, 2017; Patel et al., 2020; Patel, Vachhrajani & Trivedi, 2022). *Leptodius exaratus* (H. Milne Edwards, 1834) is a xanthid crab that is commonly found in the rocky shores of Saurashtra coast (Patel, Patel & Trivedi, 2021b). This crab species has been commonly reported from the rocky intertidal regions of Indo-Pacific region (Kneib & Weeks, 1990; Naderloo, 2017). It is an omnivorous species that prefers benthic fauna over algae to feed upon and is expected to have a considerable impact on how the benthic ecosystem is structured (Al-Wazzan et al., 2020).

In the Indian subcontinent *L. exaratus* is recorded from Andaman and Nicobar Islands, Maharashtra, Tamil Nadu, Lakshadweep Islands, Goa, Karnataka, and Gujarat (Trivedi et al., 2018). Though the species is very commonly found on the Saurashtra coast of Gujarat, studies only on its taxonomy (Chopra & Das, 1937; Trivedi & Vachhrajani, 2015) and colour variation (Patel, Patel & Trivedi, 2021b) have been carried out so far. Hence the current investigation was aimed to (1) understand the population structure and (2) study the breeding biology of *L. exaratus* occurring on the rocky shore of Saurashtra coast, Gujarat state, India in order to obtain knowledge about the ecology of rocky intertidal habitats. Studying the population structure and breeding biology of *L. exaratus* which is commonly found on the Saurashtra coast, would provide a baseline data that plays a pivotal role in understanding the effects of changing environment, habitat, or anthropogenic pressure. The present study will help in elucidating the coastal health of study area.

## Materials and Methods

### Study area

**Figure 1:**
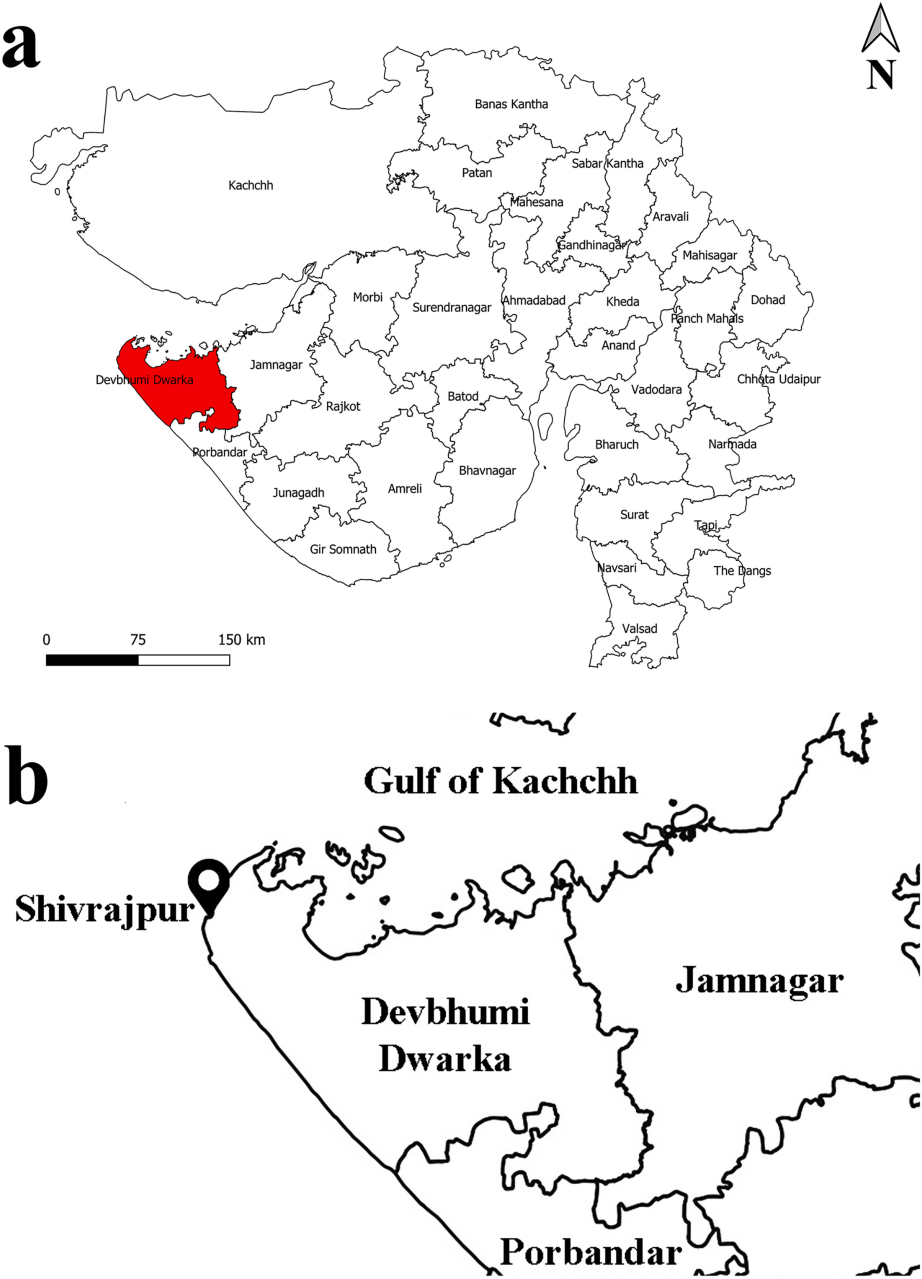
Map of study area: (A) Gujarat state; (B) Shivrajpur village, Gujarat state, India. (Prepared using QGIS version 3.14).

**Figure 2:**
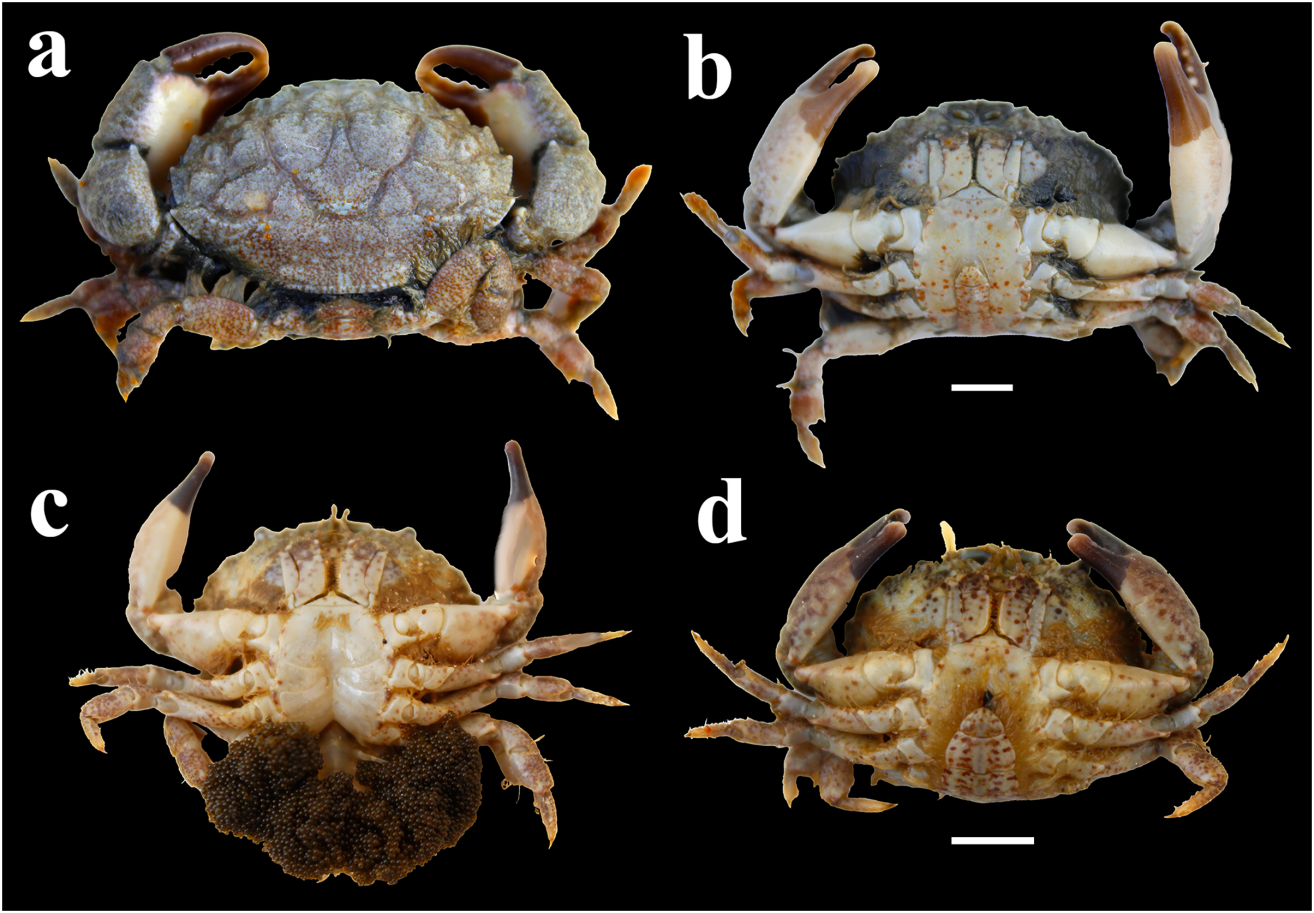
Morphology of *L. exaratus* from Shivrajpur, Gujarat state, India; (A) dorsal view; (B) ventral view male (CW: 29.98 mm); (C) ventral view ovigerous female (CW: 27.79 mm), (D) ventral view female (CW: 28.63 mm). Scale bar 5 mm.

The investigation was conducted on the rocky shore of Shivrajpur (22°19′55″N 68°57′03″E) which is located on the Saurashtra coast of Gujarat state, India (Fig. 1). *Leptodius exaratus* is a small crab found abundantly in the rocky shore of the study area (Fig. 2). During low tide, the exposed area of rocky intertidal region varies from 60 to 150 m.

### Field methods

Monthly field work was conducted for 12 consecutive months from March 2021 to February 2022. The month wise data was compiled into winter season (November to February months), summer season (March to June months), and monsoon seasons (July to October months), following Rao & Rama-Sharma (1990), to observe the seasonal variation. Catch-per-unit effort using the hand-picking method was used for the collection of specimens for a time period of 4 h at the time of low tide. When the water receded, a 500 m^2^ area in the intertidal region was marked off and thoroughly examined for the presence of *L. exaratus*. Small rocks were also upturned for the presence of *L. exaratus*, which they prefer to occupy. Whenever an individual crab was encountered, the crab was collected and preserved in 10% formalin pending additional examination.

### Laboratory analysis

The crabs were identified on the basis of their morphological characters as follows using standard identification key provided by Lee et al. (2013): Carapace is transversely sub ovate, and lightly granular. There are four large, triangular teeth on the anterolateral border, behind the exorbital angle. Male abdomen tapered, somites 3–5 fused, somite 6 elongated, 1.6 times longer than the telson. Chelipeds are unequal in size; fingers are stout with dark pigmentation excluding the tips, which are white in colour. Further, the individuals were categorised as male, non-ovigerous female, or ovigerous female (Fig. 2). For morphological character, carapace width (CW) was measured by digital vernier callipers (Mitutoyo 500-197-20) (0.01 mm accuracy) and wet weight of crabs was measured using weighing balance (Sartorius–BSA224S–CW) (0.001 g accuracy).

The following method for fecundity study was adopted from Patel, Vachhrajani & Trivedi (2023). Fecundity estimation conducted by cautiously taking out the mass of eggs present on the pleopods of ovigerous females (*n* = 34) and measuring three parameters *i.e*., total number of eggs, weight of egg mass and size of eggs (diameter). For the total number of eggs, the egg mass was transferred into 20 ml of sea water and mixed gently so that the eggs got distributed evenly in the water. From this solution, three samples of 2 ml each were taken in a petri dish and observed under a stereo zoom microscope (Matlab–PST–901; Matlab, Natick, MA, USA) to count the total number of eggs. The total number of eggs in each sample was divided by three and multiplied by the dilution factor (10) to obtain the total number of eggs (Litulo, 2004). Ovigerous females were weighed both with and without egg mass, and the difference in their weight was considered as the weight of egg mass. Eggs (*n* = 10) from each ovigerous female were measured by means of an ocular micrometre under a microscope for the size range (Saher & Qureshi, 2010).

### Data analysis

#### Population structure

The specimens were grouped in 2 mm size class intervals from 4 to 30 mm CW in order to get the overall size frequency distribution. Shapiro Wilk test was conducted to analyse the normality of the collected data, which suggests that the data distribution was not normal (*p* < 0.001). Hence, non-parametric analysis was carried out. To investigate the difference in the variance of mean values of the carapace width of male, non-ovigerous, and ovigerous individuals, a Kruskal-Wallis (KW) test was conducted. On getting a significant difference (*p* < 0.05) in the CW between the sexes, a multiple comparison analysis using Dunn’s *post hoc* test was used to do a multiple comparison study. Monthly variations in the size (CW) and sex composition of *L. exaratus* individuals were obtained by plotting the data on individuals’ carapace width and sex. The ratio of males and females (ovigerous and non-ovigerous females) was evaluated by the means of chi-square test ( $\chi^2$). The size at first maturity was determined by calculating the percentage of ovigerous females across various size classes from the total number of samples collected. Juveniles were defined as individuals that were smaller than the smallest ovigerous female (Baeza et al., 2013). The effect of temperature on *L. exaratus* breeding and juvenile settling was examined by plotting monthly data on the incidence of juvenile and ovigerous females against ambient temperature. The relationship between the mean ambient temperature and the relative juvenile frequency was examined using Pearson’s correlation analysis.

#### Fecundity

To investigate the relationship between the morphological features of eggs (total number of eggs, egg mass weight and size of eggs) and crabs’ morphology (CW and weight) regression analysis was performed. At *p* < 0.05, the statistical significance was deemed significant. Microsoft Excel and PAST software, version 4.03 (Hammer, Harper & Ryan, 2001), were used to carry out statistical analyses.

## Results

During the study period, 1,215 individuals were investigated in total; 558 of them were male (45.93%) and 657 of them were female (54.07%) (Table 1). The carapace width of *L. exaratus* males ranged from 5.15 to 29.98 mm, while in case of females it ranged from 5.26 to 28.63 mm. The size differences between the male and female individuals were not statistically significant (Kruskal-Wallis, H = 0.209, *p* = 0.646) (Table 1).

**Table 1:** Carapace width values of male and female individuals of *L. exaratus* from Shivrajpur, Gujarat state, India.

Sex	*n*	Min. CW (mm)	Max. CW (mm)	Mean ± SD
Male	558	5.15	29.98	15.967 ± 5.27*
Female	657	5.26	28.63	15.48 ± 3.77*

**Notes:**

*n*, total number of individuals; CW, carapace width.

* Significant level if *p* < 0.05 (*).

Table 2 shows that the year-round average total sex ratio (1:1.2) for *L. exaratus* was significantly different from the predicted 1:1 proportion ( $\chi^2$ = 4.1219, *p* = 0.042) and biased towards females. Month wise, female biased sex ratio was observed in almost all the months except September (1:0.8), October (1:0.9), and November (1:0.7). November had the highest percentage of male occurrences (57.89%), while April had the lowest rate (37.41%). In terms of females, the highest percentage of non-ovigerous female occurrences were observed in June (50%) and August (50%), while the lowest percentages were observed in January (27.18%). Ovigerous females were collected all year, which shows the species is breeding all year-round. However, from December to April, the greatest percentage of occurrence was recorded of ovigerous females, suggesting a peak in the breeding season.

**Table 2:** Total number of *L. exaratus* specimens collected from Shivrajpur, Gujarat state, India.

Month	M	%	NOF	%	OF	%	NOF+ OF	%	Sex ratio
January	51	49.51	28	27.18	24	23.30	52	50.49	1:1.02
February	56	43.41	40	31.01	33	25.58	73	56.59	1:1.3
March	55	44.00	42	33.60	28	22.40	70	56.00	1:1.3
April	55	37.41	51	34.69	41	27.89	92	62.59	1:1.7
May	69	40.83	69	40.83	31	18.34	100	59.17	1:1.4
June	20	43.48	23	50.00	3	6.52	26	56.52	1:1.3
July	17	45.95	14	37.84	6	16.22	20	54.05	1:1.2
August	44	44.00	50	50.00	6	6.00	56	56.00	1:1.3
September	54	56.25	37	38.54	5	5.21	42	43.75	1:0.8
October	45	53.57	34	40.48	5	5.95	39	46.43	1:0.9
November	44	57.89	22	28.95	10	13.16	32	42.11	1:0.7
December	48	46.60	32	31.07	23	22.33	55	53.40	1:1.1
Total	558	45.93	442	36.38	215	17.70	657	54.07	1:1.2

**Note:**

M, Male; NOF, Non-ovigerous female; OF, Ovigerous female. Chi-square test ( $\chi^2$) = 4.1219, *p* = 0.042.

**Figure 3:**
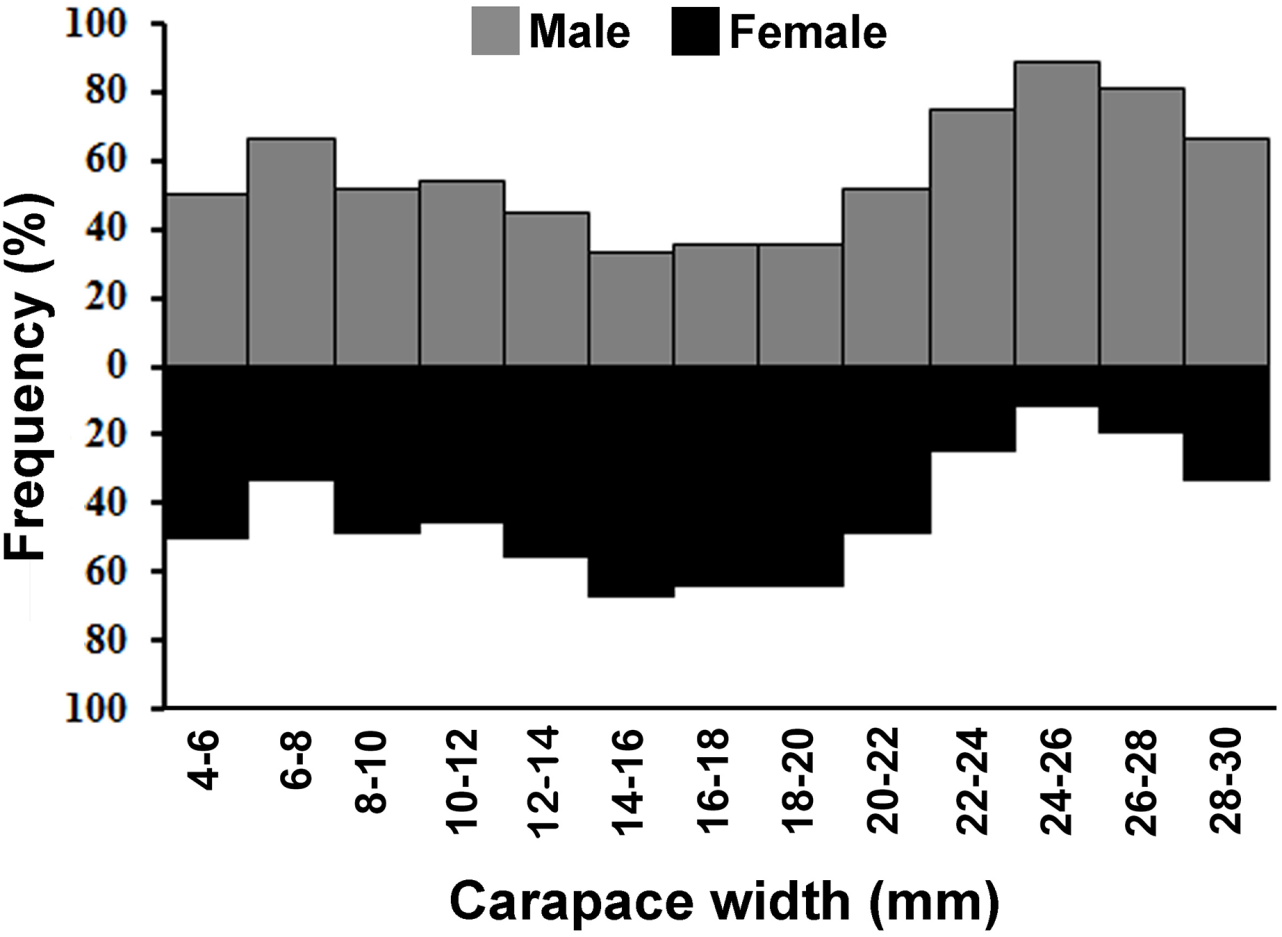
Overall size frequency distribution of *L. exaratus* collected from Shivrajpur, Gujarat state, India.

The individuals of *L. exaratus* occurred in all the size classes between 4 to 30 mm. It was observed that males exhibited bimodal pattern of distribution having maximum occurrence in 6–8 mm CW size class and 24–26 mm CW size class. On the other hand, females exhibited unimodal pattern of size frequency distribution, with maximum occurrence recorded in 14–16 mm CW size class (Fig. 3).

**Figure 4:**
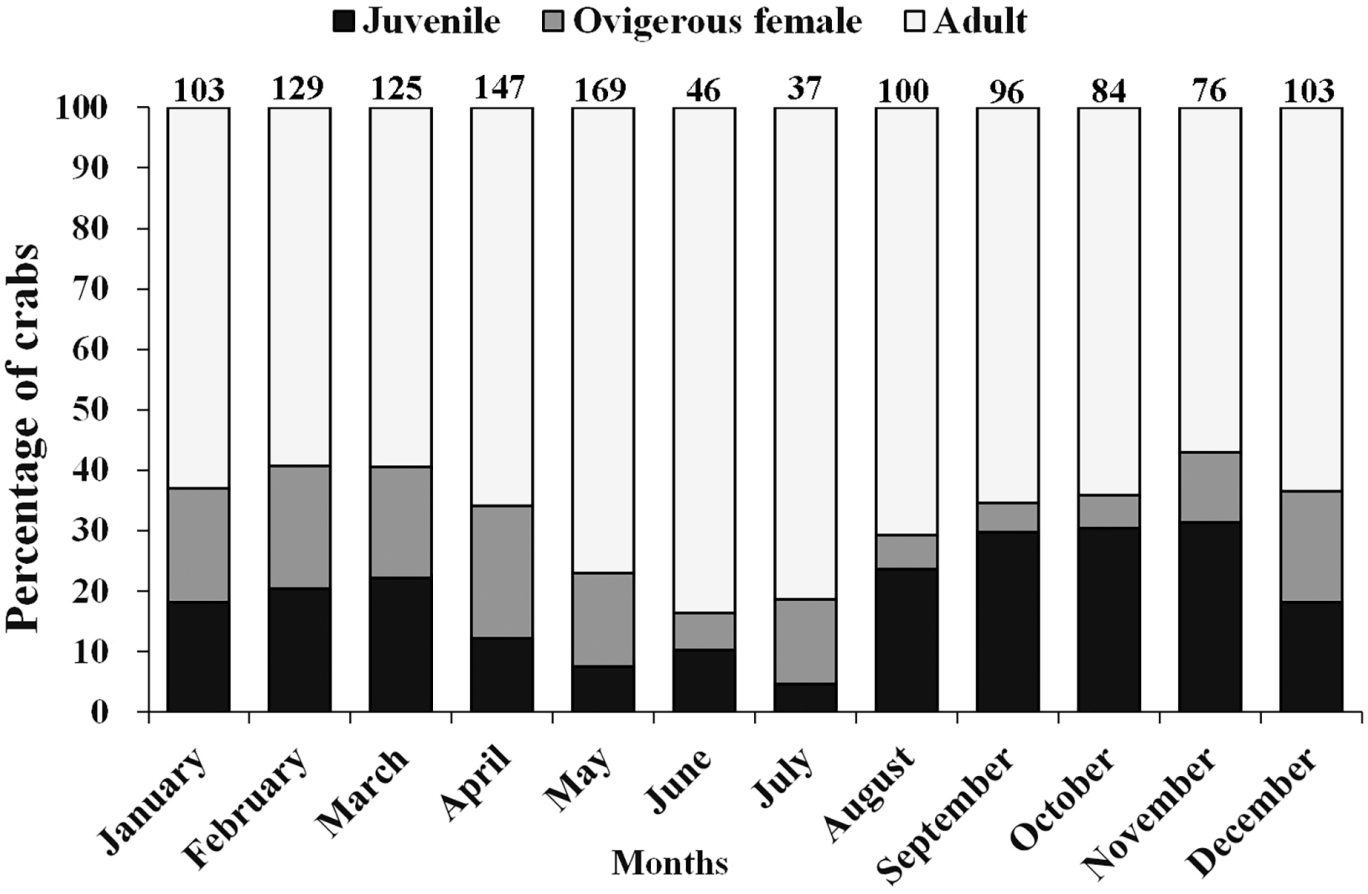
Percentage of different demographic categories of *L. exaratus* from Shivrajpur, Gujarat state, India during the 12 months of study period.

Moreover, there was a considerable variation in the occurrence of adults, ovigerous females and juveniles (<12 mm) during different months of the year (Fig. 4). It was found that in April, May, June, and July (summer and early monsoon season) the population of juveniles was least as compared to the adult population. Moderately, less number of juveniles were also observed during December to March (winter and early summer season) than August to November (Monsoon and early winter season) as compared to adult male and female (Fig. 4).

**Figure 5:**
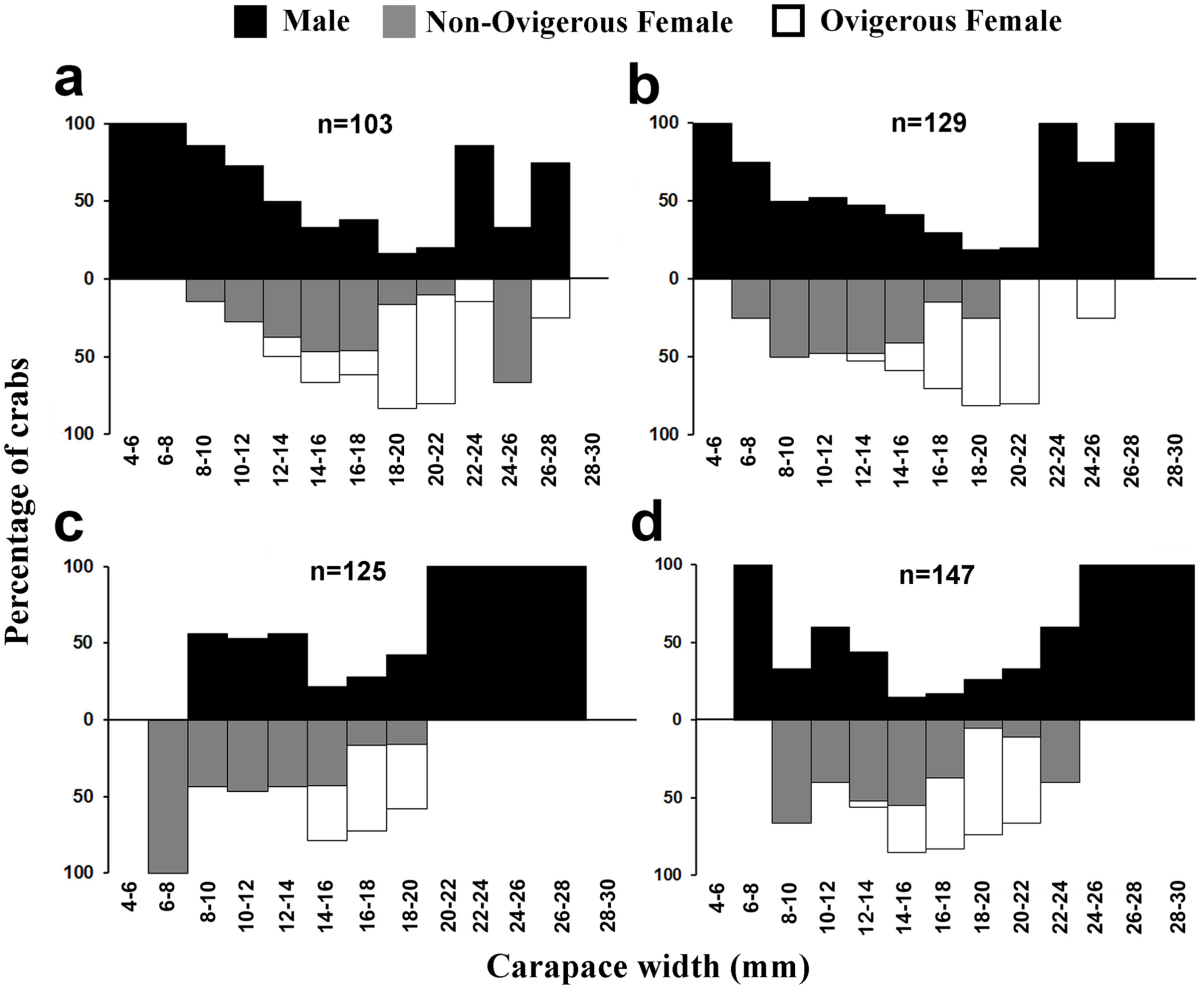
Size frequency distribution of *L. exaratus* in each month from Shivrajpur, Gujarat state, India; (A) January, (B) February, (C) March, (D) April.

**Figure 6:**
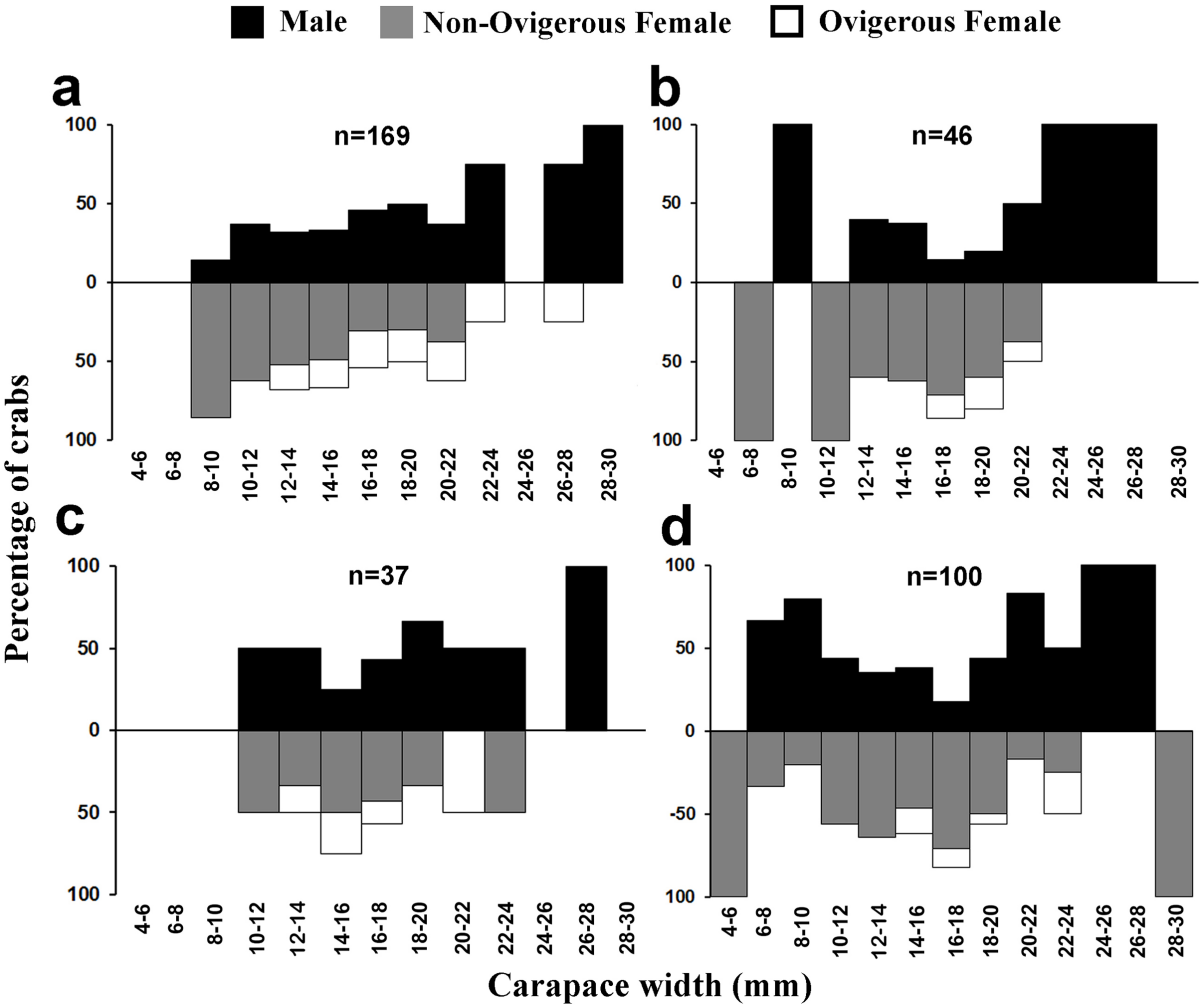
Size frequency distribution of *L. exaratus* in each month from Shivrajpur, Gujarat state, India; (A) May, (B) June, (C) July, (D) August.

**Figure 7:**
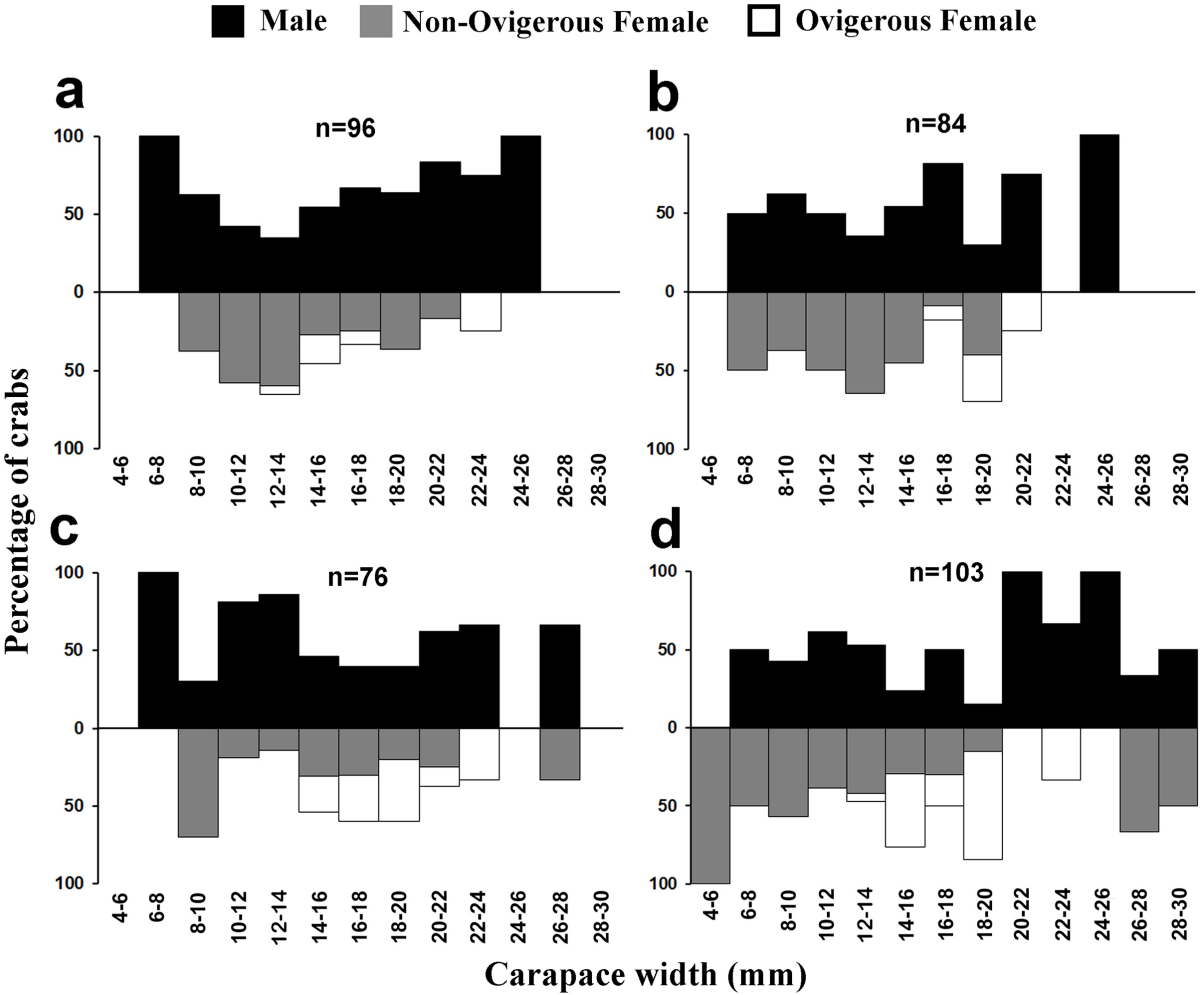
Size frequency distribution of *L*. exaratus in each month from Shivrajpur, Gujarat state, India; (A) September, (B) October, (C) November, (D) December.

**Figure 8:**
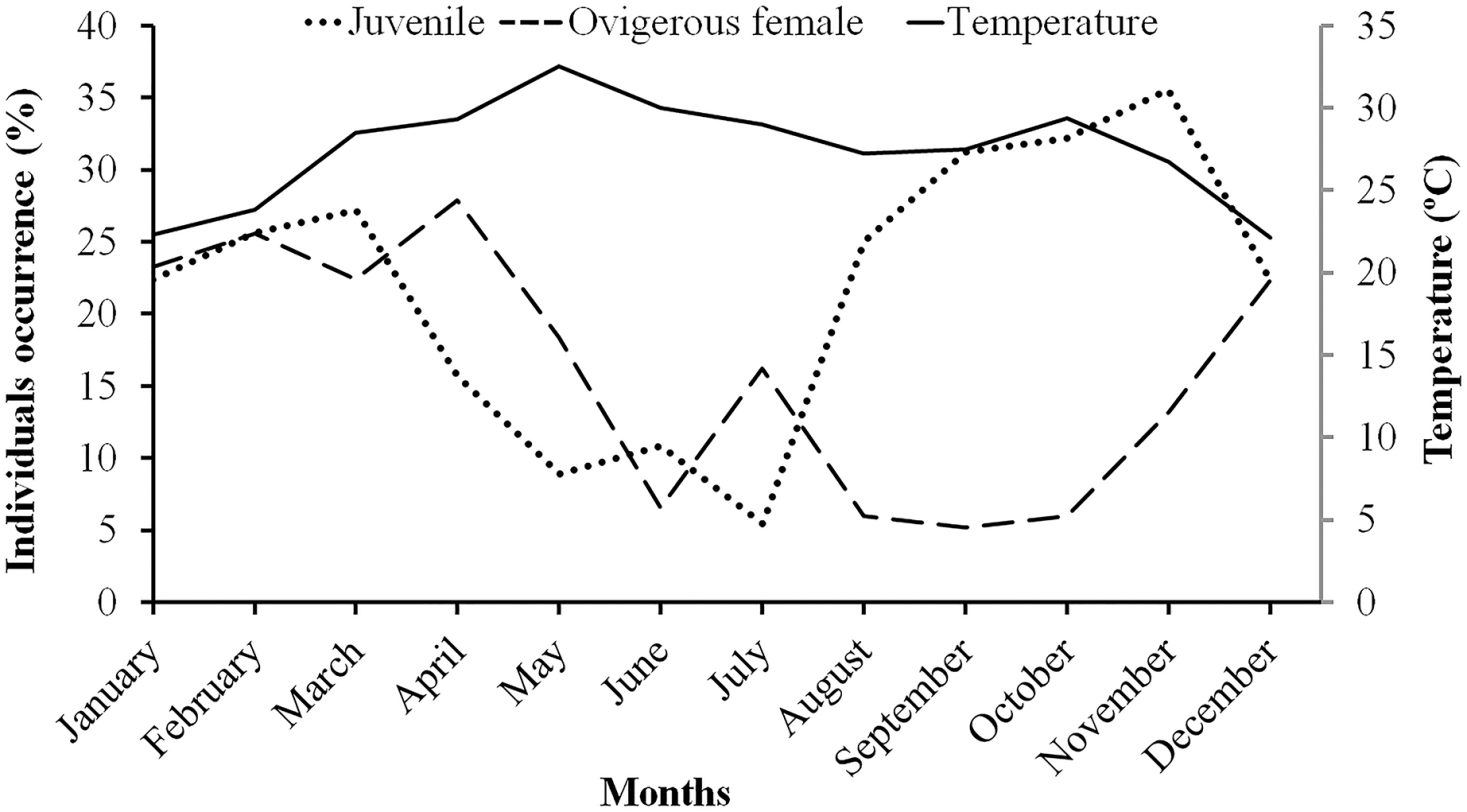
Association between the juveniles (of both sexes) and ovigerous female occurrence of *L. exaratus* with monthly ambient temperature from Shivrajpur, Gujarat state, India.

Males had a bimodal distribution during most of the months, while non-ovigerous females showed a unimodal distribution pattern, as was also observed in ovigerous females. Furthermore, it was also observed that juveniles were present all year round (Figs. 5–7). A negative correlation (Pearson’s correlation, r = −0.39) was observed between the mean ambient temperature and relative frequency of juveniles (Fig. 8).

**Figure 9:**
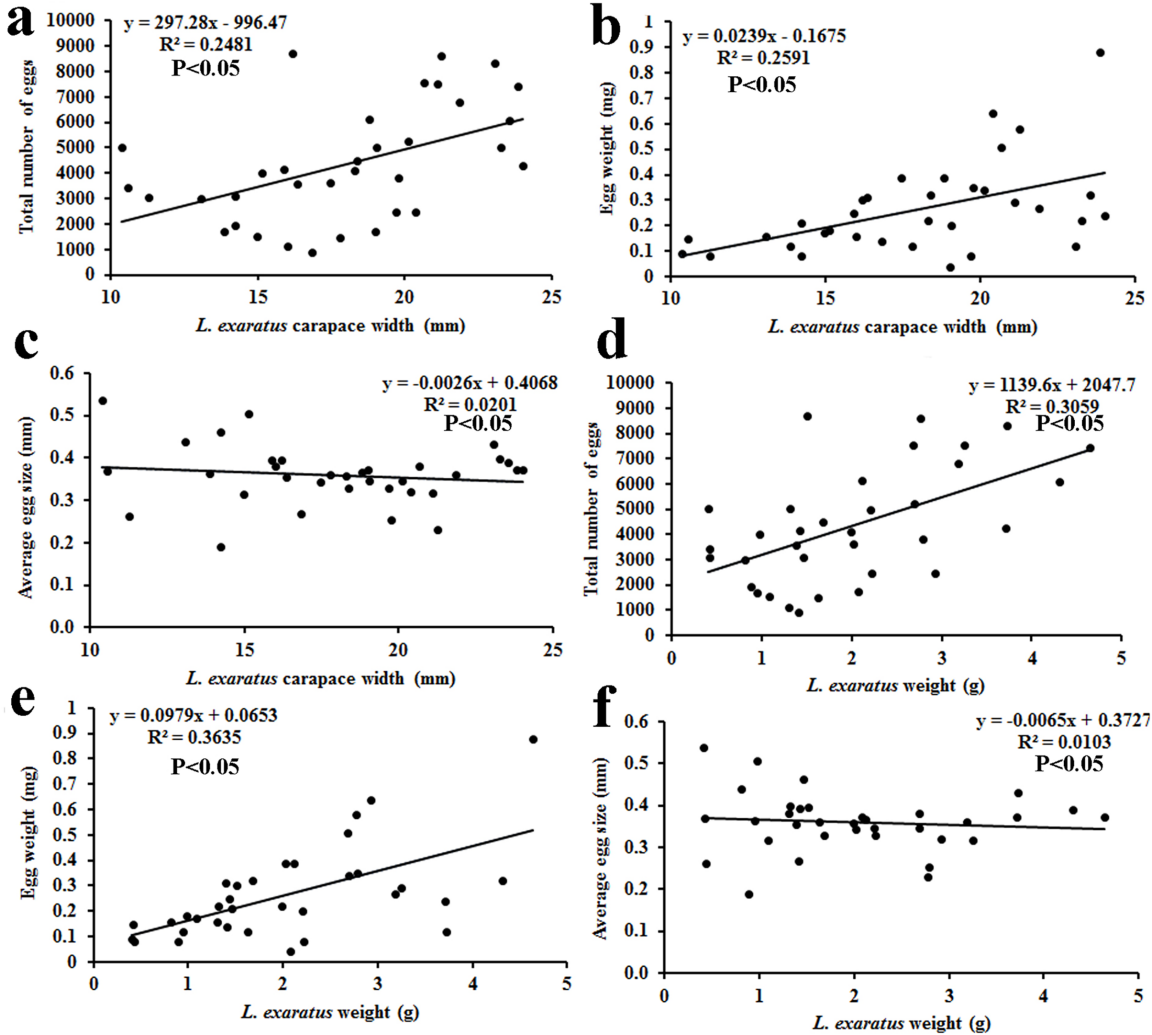
Relationship of *L. exaratus* carapace width (mm) with (A) total number of eggs; (B) egg weight; and (C) average egg size; and crab weight (g) with (D) total number of eggs; (E) egg weight; and (F) average egg size.

The results of fecundity revealed that the CW of ovigerous females was between 10.38 and 24.02 mm, with their average size being 17.95 ± 3.81 mm (*n* = 34). The wet body weight of the ovigerous females was recorded between 0.41 and 4.64 g, with the mean weight being 2.01 ± 1.1 g (*n* = 34). The average number of eggs observed was 4,529 ± 2,003 (*n* = 34), with the minimum and maximum reported being 920 and 8,730 eggs, respectively. The average egg size (*n* = 34) was 0.36 ± 0.07 mm, with the minimum and maximum observed sizes being 0.19 and 0.54 mm, respectively. The average egg mass weight (*n* = 34) was 0.29 ± 0.18 g, with the minimum and maximum observed egg mass weights being 0.04 and 0.88 g, respectively (Table 3). The ovigerous females’ carapace width and body weight were shown to be significantly correlated with both the egg weight and total number of eggs (Fig. 9).

**Table 3:** Summary of different morphological parameters of *L. exaratus* ovigerous females and eggs from Shivrajpur, Gujarat state, India.

Variables	*n*	Mean ± SD	Min.	Max.
Crab weight (g)	34	2.04 ± 1.2	0.41	4.64
Weight of egg mass (g)	34	0.29 ± 0.18	0.04	0.88
Carapace length (mm)	34	12.14 ± 2.4	6.92	15.88
Carapace width (mm)	34	18.1 ± 3.8	10.38	24.02
Egg number	34	4,529 ± 2,003	920	8,730
Egg size (mm)	34	0.36 ± 0.07	0.19	0.54

**Note:**

*n*, total individuals; SD, standard deviation.

## Discussion

A significant variation was observed in the average carapace width of different sexes of *Dotilla blanfordi*, where it was found that male individuals were significantly larger than females. Studies conducted on the population structure of *Matuta planipes* and *Ashtoret lunaris* (Saher et al., 2017), *Uca bengali* (Tina et al., 2015), *Scylla olivacea*, *S*. *tranquebarica*, and *S*. *paramamosain* (Waiho et al., 2021), *Clibanarius ransoni* (Patel, Patel & Trivedi, 2020; Patel, Vachhrajani & Trivedi, 2022), *C. rhabdodactylus* (Patel, Vachhrajani & Trivedi, 2023), and *Diogenes custos* (Patel et al., 2020) also exhibited similar results. It has been observed that the growth rate of female individuals is generally reduced as a result of greater energy investment in gonadal development, which leads to decreased somatic growth in comparison to male individuals (Mantelatto et al., 2010). Another hypothesis suggests that the chances of attracting and obtaining females for the purpose of mating increases with increased size of male individuals (Wada, Kitaoka & Goshima, 2000), while the difference in size also reduces intraspecific competition among different sexes for available resources (Abrams, 1988).

Overall sex ratio (1:1.2) was found to be female-biased, while month-wise also female biased sex ratio was observed except September, October and November months. In general, natural selection promotes a sex ratio of 1:1 parental expenditure on offspring (Taylor, 1996); however, deviation from the ideal sex ratios is common in marine crustaceans, as observed in *Calcinus tibicen* (Fransozo & Mantelatto, 1998), *Limulus polyphemus* (Smith et al., 2002), *Crangon crangon* (Siegel, Damm & Neudecker, 2008), *Opusia indica* (Saher & Qureshi, 2011), and *Macrophthalmus* (*Venitus*) *dentipes* (Qureshi & Saher, 2012). The sex ratio also differed during different growth stages, with an ideal sex ratio (1:1) in smaller size classes (1–3 mm CW), female biased in intermediate size classes (3–6 mm CW) and exclusively male biased in larger individuals (6–8 mm CW). Certain other studies have found similar results (Gherardi & Nardone, 1997; Bezerra & Matthews-Cascon, 2007; Mishima & Henmi, 2008; Manzoor et al., 2016). Numerous factors can be responsible for such deviation in the sex ratio including competition in local mate (Hamilton, 1967), differences in the efficiency of utilizing local resources that biases sex ratios (Silk, 1983), difference in the investment in male and female offspring (Kobayashi et al., 2018), and sexual selection (Swanson et al., 2013). Sexual dimorphism in size could be one of the reasons for the different sex ratio from the ideal 1:1 in different size classes. Higher male mortality in the intermediate-size classes often leads to a female biased sex ratio (Asakura, 1995). Moreover, males grow to bigger sizes quickly than females, leading to male biased sex ratio on the larger size classes (Wenner, 1972). Disparities in sexual mortality and dispersion may potentially contribute to unbalanced sex ratios in crab populations (Johnson, 2003).

Present investigation found that the size frequency distribution of *L. exaratus* males had a bimodal distribution, while the females had a unimodal distribution. Also, there was a considerable difference in the seasonal size frequency distribution. Similar results have been observed in *Paguristes tortugae* (Mantelatto & Sousa, 2000), *Chaceon affinis* (López Abellán, Balguerías & Fernández-Vergaz, 2002), *Pilumnus vespertilio* (Litulo, 2005), *Dilocarcinus pagei* (Taddei et al., 2015) *Aegla georginae* (Copatti et al., 2016) and *Clibanarius rhabdodactylus* (Patel, Vachhrajani & Trivedi, 2023). Over time, the population size and frequency of dispersion may be significantly changed by the rapid recruitment of larvae and reproductive rate (Thurman, 1985). Such distributions have been explained by a variety of theories, such as differential patterns of migration (Flores & Negreiros-Fransozo, 1999), growth rate (Negreiros-Fransozo, Costa & Colpo, 2003), and differential death rate (Díaz & Conde, 1989). It is commonly found in organisms that undergo several rounds of reproduction and generate a large number of clutches every season (Zimmerman & Felder, 1991). Unimodality is often seen in stable populations that have approximately equal numbers of new members and emigrants, consistent recruitment and mortality rates throughout the course of the life cycle, and steady demographics (Thurman, 1985; Díaz & Conde, 1989) whereas, bimodality could be an indication of the general tendencies in population increase.

The ambient temperature of the study site ranged from 22.1 °C to 32.5 °C, which is within the range of a tropical environment that may support continuous reproduction. Hence, there was year-round occurrence of ovigerous females suggesting that *L. exaratus* is a continuously breeding species that has maximum recorded frequency from December to April. Similarly, studies carried out on *L. exaratus* (Al-Wazzan et al., 2020), *Scylla olivacea* (Ali et al., 2020), *Opusia indica* (Saher & Qureshi, 2011), *Emerita portoricensis* and *E. asiatica* (Goodbody, 1965), *Ilyoplax frater* (Saher & Qureshi, 2010), *Diogenes brevirostris* (Litulo, 2004) and *Petrochirus diogenes* (Bertini & Fransozo, 2002) did not find any association between the frequency occurrence of ovigerous females and ambient temperature. As *L. exaratus* is common inhabitant of upper intertidal region where higher temperature during summer season can greatly increase the desiccation risk, leading to migration of ovigerous females in deeper water (Allen, 1966; Asakura, 1987; Al-Wazzan et al., 2020) resulting in decreased abundance in the intertidal region. As a result, seasonal fluctuations in abundance reflect both migration and mortality, while summer abundance estimates may underestimate the size of the local population. However, it was found that juvenile percentage occurrence increased with a decline in ovigerous female percentage occurrence, whereas juvenile percentage occurrence declined when ovigerous female percentage occurrence increased. Such outcomes demonstrate that the species may recruit juveniles throughout the year as a result of rapid reproduction and a short incubation time. Similar outcomes have been reported in several other studies including *Deiratonotus japonicus* (Oh & Lee, 2020), *Scylla olivacea* (Rouf et al., 2021), *Clibanarius rhabdodactylus* (Patel, Vachhrajani & Trivedi, 2023), *Dardanus deformis* (Litulo, 2005), and *Menippe nodifrons* (Fransozo, Bertini & Correa, 2000). There are a number of variables, including the availability of food for adults (Goodbody, 1965), the ecology of larvae (Reese, 1968), the amount of time to attain sexual maturity, the timing of mating and gonadal development, as well as the length of the incubation period (Sastry, Vernberg & Vernberg, 1983), which can lead to periodicity in the reproductive season. A variety of abiotic and biotic variables, including water temperature (Chou, Head & Backwell, 2019), salinity (Huang et al., 2022), the nutritional quality of the females (Matias et al., 2016), variations in photoperiod (Zhang et al., 2023), the amount and availability of nutrition (ViñaTrillos, Brante & Urzúa, 2023), and the threat of predation (Touchon, Gomez-Mestre & Warkentin, 2006), may affect the reproductive maxima among populations.

It was found that the CW and wet body weight of *L. exaratus* were having positive correlation with total number of eggs and egg mass weight. Several other studies have also found similar results (Patel, Vachhrajani & Trivedi, 2023; Crowley et al., 2019; Hamasaki, Ishii & Dan, 2021; Aviz et al., 2022; Mustaquim, Khatoon & Rashid, 2022). Additionally, it has been demonstrated that ovigerous females with the same CW had variations in the number of eggs, egg mass weight, and egg size resulting from variations in the food supply, variation in egg production, and egg loss (Hines, 1982).

## Conclusions

The goal of the current study was to better understand the population structure and breeding biology of *L. exaratus*. Significant sexual dimorphism was found, with males being larger than females, most likely as a result of the size of gamete formation differing between the sexes and females investing more in egg production. Total sex ratio of species was 1:1.2 and monthly populations may be a result of differential biology and behaviour as well as the impact of biotic and abiotic variables on male and female individuals. The year-round occurrence of ovigerous females suggests continuous breeding of the population and an inverse relationship between the peak in juvenile recruitment and the occurrence of ovigerous females which is a common phenomenon of tropical brachyuran crabs. There was a positive correlation between the egg parameters (weight of egg mass and number of eggs) and the morphology of ovigerous females (carapace width and body weight). Fecundity may be impacted by a variety of internal and external variables, such as the amount of energy used for somatic development and egg production. The present study was conducted at Shivrajpur village, a renowned tourist site with a blue-flag beach where various water sports activities take place. These activities along with higher tourist rush at the study site that may impact the habitat composition of the coast and also potentially influencing the ecology of *L. exaratus*. Furthermore, our findings will contribute to understanding the species’ response to environmental changes, as both population structure and fecundity are closely tied to environmental variables.

## Supplemental Information

10.7717/peerj.16916/supp-1 Supplemental Information 1 Raw data.

## Additional Information and Declarations

### Data Availability

The following information was supplied regarding data availability: The raw data used for analysis are available in the Supplemental File.

## References

[ref-1] Abrams PA, Chelazzi G, Vannini M (1988). Sexual difference in resource use in hermit crabs; consequences and causes. Behavioral Adaptation to Intertidal Life.

[ref-2] Al-Wazzan Z, Vay LL, Behbehani M, Giménez L (2020). Scale-dependent spatial and temporal patterns of abundance and population structure of the xanthid crab *Leptodius exaratus* on rocky shores in Kuwait. Regional Studies in Marine Science.

[ref-3] Ali MY, Hossain MB, Sana S, Rouf MA, Yasmin S, Sarower MG (2020). Identifying peak breeding season and estimating size at first maturity of mud crab (*Scylla olivacea*) from a coastal region of Bangladesh. Heliyon.

[ref-4] Allen JA (1966). The rhythms and population dynamics of decapod Crustacea. Oceanography and Marine Biology.

[ref-5] Asakura A (1987). Preliminary observations on the offshore mass migration of the sand-dwelling hermit crab, *Diogenes nitidimanus* terao (Crustacea decapoda: Anomura). Journal of Ethology.

[ref-6] Asakura A (1995). Sexual differences in life history and resource utilization by the hermit crab. Ecology.

[ref-7] Aviz D, Amorim Carmona P, Caroline de Castro Barbosa A, Rannieri Meira dos Santos C (2022). Fecundity and reproductive patterns of the fiddler crab *Uca maracoani* Latreille 1802–1803 in an Amazonian estuary in northern Brazil. Invertebrate Reproduction & Development.

[ref-8] Baeza JA, Furlan M, Almeida AC, de Barros-Alves SP, Alves DFR, Fransozo V (2013). Population dynamics and reproductive traits of the ornamental crab *Porcellana sayana*: implications for fishery management and aquaculture. Sexuality and Early Development in Aquatic Organisms.

[ref-9] Bertini G, Fransozo A, Escobar-Briones E, Alvarez F (2002). Breeding season of the hermit crab *Petrochirus diogenes* (Anomura: Diogenidae) in the north coast of São Paulo State, Brazil. Modern Approaches to the Study of Crustacea.

[ref-10] Bezerra LEA, Matthews-Cascon H (2007). Population and reproductive biology of the fiddler crab *Uca thayeri* Rathbun, 1900 (Crustacea: Ocypodidae) in a tropical mangrove from Northeast Brazil. Acta Oecologica.

[ref-11] Bhat M, Trivedi J (2021). New record of the Sesarmid crab *Episesarma versicolor* (Tweedie, 1940) (Crustacea: Decapoda: Sesarmidae) from the West Coast of India. Journal of Biological Studies.

[ref-12] Chopra BN, Das KN (1937). Further notes on Crustacea Decapoda in the Indian Museum. IX. On three collections of crabs from Tavoy and Mergui Archipelago. Records of the Indian Museum.

[ref-13] Chou C-C, Head ML, Backwell PRY (2019). Effects of temperature on reproductive timing and hatching success in a tropical fiddler crab. Biological Journal of the Linnean Society.

[ref-14] Copatti CE, Legramanti RP, Trevisan A, Santos S (2016). Growth, sexual maturity and sexual dimorphism of *Aegla georginae* (Decapoda: Anomura: Aeglidae) in a tributary of the Ibicuí River in southern Brazil. Zoologia (Curitiba).

[ref-15] Crowley CE, Shea CP, Gandy RL, Daly KL (2019). Fecundity assessment of stone crabs in the Eastern Gulf of Mexico. Marine and Coastal Fisheries.

[ref-16] Díaz H, Conde JE (1989). Population dynamics and life history of the mangrove crab *Aratus pisonii* (Brachyura, Grapsidae) in a marine environment. Bulletin of Marine Science.

[ref-17] Flores A, Negreiros-Fransozo ML (1999). Allometry of the secondary sexual characters of the shore crab *Pachygrapsus transversus* (Gibbes, 1850) (Brachyura, Grapsidae). Crustaceana.

[ref-18] Flores AAV, Paula J (2002). Population dynamics of the shore crab *Pachygrapsus marmoratus* (Brachyura: Grapsidae) in the central Portuguese coast. Journal of the Marine Biological Association of the United Kingdom.

[ref-19] Fransozo A, Bertini G, Correa M (2000). Population biology and habitat utilization of the stone crab *Menippe nodifrons* in the Ubatuba region, São Paulo, Brazil. Biodiversity Crisis and Crustacea.

[ref-20] Fransozo A, Mantelatto FLM (1998). Population structure and reproductive period of the tropical hermit crab *Calcinus tibicen* (Decapoda: Diogenidae) in the region of Ubatuba, São Paulo, Brazil. Journal of Crustacean Biology.

[ref-21] Gherardi F, Nardone F (1997). The question of coexistence in hermit crabs: population ecology of a tropical intertidal assemblage. Crustaceana.

[ref-22] Goodbody I (1965). Continuous breeding in populations of two tropical crustaceans, *Mysidium columbiae* (Zimmer) and *Emerita portoricensis* Schmidt. Ecology.

[ref-23] Gosavi S, Purohit B, Mitra S, Patel K, Vachhrajani K, Trivedi J (2021). Annotated checklist of marine decapods (Crustacea: Decapoda) of Gujarat state with three new records.

[ref-24] Gosavi S, Trivedi J, Trivedi D, Vachhrajani K (2017a). First record of *Leucisca squalina* MacLeay, 1838 (Decapoda: Leucosiidae) from Gujarat state, India. Journal of Entomology and Zoology Studies.

[ref-25] Gosavi S, Trivedi J, Trivedi D, Vachhrajani K (2017b). New records of Anomuran crabs (Crustacea: Decapoda: Anomura) from Gujarat, India. Journal of Entomology and Zoology Studies.

[ref-26] Hamasaki K, Ishii M, Dan S (2021). Seasonal variability in fecundity and egg size in the porcellanid crab *Petrolisthes japonicus* (Decapoda: Anomura: Porcellanidae). Crustacean Research.

[ref-27] Hamilton WD (1967). Extraordinary sex ratios. Science.

[ref-28] Hammer Ø, Harper DAT, Ryan PD (2001). PAST-palaeontological statistics. Palaeontologia Electronica.

[ref-29] Hines AH (1982). Allometric constraints and variables of reproductive effort in brachyuran crabs. Marine Biology.

[ref-30] Hu MPK, Kwan B, Wang Y, Cheung S, Shin P, Carmichael R, Botton M, Shin P, Cheung S (2015). Population structure and growth of juvenile horseshoe crabs *Tachypleus tridentatus* and *Carcinoscorpius rotundicauda* (Xiphosura) in Southern China. Changing Global Perspectives on Horseshoe Crab Biology, Conservation and Management.

[ref-31] Huang X, He L, Tan R, Feng G, Geng Z, Zhao F, Zhang T, Zhuang P (2022). Effects of salinity on reproductive characteristics and embryo quality of *Eriocheir sinensis*. Aquaculture Research.

[ref-32] Johnson P (2003). Biased sex ratios in fiddler crabs (Brachyura, Ocypodidae): a review and evaluation of the influence of sampling method, size class, and sex-specific mortality. Crustaceana.

[ref-33] Kneib RT, Weeks CA (1990). Intertidal distribution and feeding habits of the mud crab *Eurytium limosum*. Estuaries.

[ref-34] Kobayashi M, Wong YH, OguroOkano M, Dreyer N, Høeg JT, Yoshida R, Okano K (2018). Identification, characterization, and larval biology of a rhizocephalan barnacle, *Sacculina yatsui* Boschma, 1936, from northwestern Japan (Cirripedia: Sacculinidae). Journal of Crustacean Biology.

[ref-35] Lee SK, Mendoza JCE, Ng PKL, Kim W (2013). On the identity of the indo-west pacific littoral xanthid crab, *Leptodius exaratus* (H. Milne Edwards, 1834) (crustacea: decapoda: brachyura: xanthidae). Raffles Bulletin of Zoology.

[ref-36] Litulo C (2004). Fecundity of the pantropical fiddler crab *Uca annulipes* (H. Milne Edwards, 1837) (Brachyura: Ocypodidae) at Costa do Sol mangrove, Maputo bay, Southern Mozambique. Western Indian Ocean Journal of Marine Science.

[ref-37] Litulo C (2005). Population structure and breeding biology of the hairy crab *Pilumnus vespertilio* (Fabricius, 1793) (Crustacea: Brachyura: Pilumnidae) in southern Mozambique. Journal of Natural History.

[ref-39] López Abellán LJ, Balguerías E, Fernández-Vergaz V (2002). Life history characteristics of the deep-sea crab *Chaceon affinis* population off Tenerife (Canary Islands). Fisheries Research.

[ref-38] Lycett KA, Shields JD, Chung JS, Pitula JS (2020). Population structure of the blue crab *Callinectes sapidus* in the Maryland Coastal Bays. Journal of Shellfish Research.

[ref-40] Mantelatto FL, Fernandes-Góes LC, Fantucci MZ, Biagi R, Pardo LM, Marcos de Góes J (2010). A comparative study of population traits between two South American populations of the striped-legged hermit crab *Clibanarius vittatus*. Acta Oecologica.

[ref-41] Mantelatto FLM, Sousa LM (2000). Population biology of the hermit crab *Paguristes tortugae* Schmitt, 1933 (Anomura, Diogenidae) from Anchieta Island. Nauplius.

[ref-42] Manzoor R, Haider S, Fatima M, Qari R (2016). Study on abundance, breeding and growth of the Ocypodide crab *Dotilla blanfordi* in Karachi Coast, Pakistan. International Journal of Marine Science.

[ref-43] Matias D, Joaquim S, Matias AM, Leitão A (2016). Reproductive effort of the European clam *Ruditapes decussatus* (Linnaeus, 1758): influence of different diets and temperatures. Invertebrate Reproduction & Development.

[ref-44] Mishima S, Henmi Y (2008). Reproduction and embryonic diapause in the hermit crab *Pagurus nigrofascia*. Crustacean Research.

[ref-45] Mustaquim J, Khatoon S, Rashid S (2022). A note on sex ratio, size at maturity, fecundity and breeding season of the portunid crab, *Thalamita crenata* Rüppell, 1830 from the Pakistani coast. Crustaceana.

[ref-46] Naderloo R (2017). Atlas of Crabs of the Persian Gulf.

[ref-47] Negreiros-Fransozo ML, Costa TM, Colpo KD (2003). Allometric growth in the fiddler crab *Uca thayeri* (Brachyura, Ocypodidae) from a subtropical mangrove. Journal of Crustacean Biology.

[ref-48] Oh I-K, Lee S (2020). Effects of temperature on the survival and larval development of Deiratonotus japonicus (Brachyura, Camptandriidae) as a biological indicator. Journal of Marine Science and Engineering.

[ref-49] Padate VP, Patel KJ, Rivonker CU, Trivedi JN (2022). On Indian species of *Nanosesarma* Tweedie, 1950 (Decapoda: Brachyura: Sesarmidae). Nauplius.

[ref-50] Patel D, Patel K, Patel P, Trivedi J (2020). Shell utilization pattern by the hermit crab *Diogenes custos* (Fabricius, 1798) along Gulf of Kachchh, Gujarat, India. Journal of Biological Studies.

[ref-51] Patel P, Patel K, Trivedi J (2020). First record of Hermit crab *Clibanarius ransoni* Forest, 1953 (Crustacea: Anomura: Diogenidae) from India. Journal of Biological Studies.

[ref-52] Patel K, Patel P, Trivedi J (2021a). First record of *Manningis arabicum* (Jones and Clayton, 1983) (Decapoda, Brachyura, Camptandriidae) from India. Nauplius.

[ref-53] Patel H, Patel K, Trivedi J (2021b). Study of colour variation in intertidal crab *Leptodius exaratus* (H. Milne Edwards, 1834) inhabiting rocky shores of Saurashtra coast, Gujarat, India.

[ref-54] Patel KJ, Vachhrajani KD, Trivedi J (2022). Study on shell utilization pattern of two sympatric hermit crab species on the rocky intertidal region of Veraval, Gujarat, India. Thalassas: An International Journal of Marine Sciences.

[ref-55] Patel KJ, Vachhrajani KD, Trivedi J (2023). Population structure and reproductive biology of *Clibanarius rhabdodactylus* Forest, 1953 (Crustacea: Anomura: Diogenidae) in Gujarat state, India. Regional Studies in Marine Science.

[ref-56] Qureshi NA, Saher NU (2012). Density, distribution and population biology of *Macrophthalmus* (*Venitus*) *dentipes* Lucas, 1836, from Mangrove areas of Pakistan. Pakistan Journal of Zoology.

[ref-57] Rao GS, Rama-Sharma DV (1990). Meiobenthos of the Gosthani estuary. Indian Journal of Marine Sciences.

[ref-58] Reese ES (1968). Annual breeding seasons of three sympatric species of tropical intertidal hermit crabs, with a discussion of factors controlling breeding. Journal of Experimental Marine Biology and Ecology.

[ref-59] Rouf MA, Shahriar SIM, Antu A-H, Siddiqui MN (2021). Population parameters of the orange mud crab *Scylla olivacea* (Herbst, 1796) from the Sundarban mangrove forest in Bangladesh. Heliyon.

[ref-60] Saher NU, Amanat Z, Gondal MA, Qureshi NA (2017). Distribution, abundance and population ecology of *Ashtoret lunaris* (Forskel, 1775) and *Matuta planipes* Fabricius, 1798 from the Sonmiani Bay (Lagoon), Pakistan. Pakistan Journal of Zoology.

[ref-61] Saher NU, Qureshi NA (2010). Zonal distribution and population biology of *Ilyoplax frater* (Brachyura: Ocypodoidea: Dotillidae) in a coastal mudflat of Pakistan. Current Zoology.

[ref-62] Saher NU, Qureshi NA (2011). Density, distribution and population structure of *Opusia indica* (Ocypodoidae: Camptandriidae) in a coastal mangrove creek in Pakistan. Biologia.

[ref-63] Santos S, Negreiros-Fransozo ML, Padovani CR (1995). Relação do peso em função da largura da carapaça do siri candeias *Portunus spinimanus* Latreille, 1819 (Decapoda Portunidae). Arquivos de Biologia e Tecnologia.

[ref-64] Sastry A, Vernberg FJ, Vernberg W, Vernberg FJ (1983). Ecological aspects of reproduction. Environmental Adaptations.

[ref-65] Siegel V, Damm U, Neudecker T (2008). Sex-ratio, seasonality and long-term variation in maturation and spawning of the brown shrimp *Crangon crangon* (L.) in the German Bight (North Sea). Helgoland Marine Research.

[ref-66] Silk JB (1983). Local resource competition and the evolution of male-biased sex ratios. Journal of Theoretical Biology.

[ref-67] Smith DR, Pooler PS, Swan BL, Michels SF, Hall WR, Himchak PJ, Millard MJ (2002). Spatial and temporal distribution of horseshoe crab (*Limulus polyphemus*) spawning in Delaware Bay: implications for monitoring. Estuaries.

[ref-68] Swanson BO, George MN, Anderson SP, Christy JH (2013). Evolutionary variation in the mechanics of fiddler crab claws. BMC Evolutionary Biology.

[ref-69] Taddei FG, Davanso TM, Castiglioni L, Herrera DR, Fransozo A, Caetano R (2015). Population structure, recruitment, and mortality of the freshwater crab *Dilocarcinus pagei* Stimpson, 1861 (Brachyura, Trichodactylidae) in Southeastern Brazil. Invertebrate Reproduction & Development.

[ref-70] Takween W, Qureshi N (2005). Population structure and reproductive biology of four species of swimming crabs (Crustacea: Brachyura: Portunidae) from coastal area of Karachi, Pakistan. Pakistan Journal of Marine Sciences.

[ref-71] Taylor DR (1996). Parental expenditure and offspring sex ratios in the dioecious plant *Silene alba* (= *Silene latifolia*). The American Naturalist.

[ref-72] Thurman CL (1985). Reproductive biology and population structure of the fiddler crab *Uca subcylindrical* (stimpson). The Biological Bulletin.

[ref-73] Tina FW, Jaroensutasinee M, Sutthakiet O, Jaroensutasinee K (2015). The fiddler crab, *Uca bengali* Crane, 1975: population biology and burrow characteristics on a riverbank in southern Thailand. Crustaceana.

[ref-74] Touchon JC, Gomez-Mestre I, Warkentin KM (2006). Hatching plasticity in two temperate anurans: responses to a pathogen and predation cues. Canadian Journal of Zoology.

[ref-76] Trivedi J, Mitra S, Patel P, Gosavi S, Vachhrajani K (2020). Four new records of brachyuran crabs (Crustacea: Decapoda: Brachyura) from India. Thalassas: An International Journal of Marine Sciences.

[ref-77] Trivedi J, Mitra S, Patel P, Maheta N, Patel K, Ng PKL (2021). On the Indian species of *Eurycarcinus* A. Milne-Edwards, 1867, Heteropanope Stimpson, 1858, and Pilumnopeus A. Milne-Edwards, 1867 (Decapoda: Brachyura: Pilumnidae). Nauplius.

[ref-81] Trivedi JN, Soni GM, Trivedi DJ, Vachhrajani KD (2015). A new species of *Ilyoplax* (Decapoda, Brachyura, Dotillidae) from Gujarat, India. Journal of Asia-Pacific Biodiversity.

[ref-82] Trivedi JN, Trivedi DJ, Vachhrajani KD, Ng PKL (2018). An annotated checklist of the marine brachyuran crabs (Crustacea: Decapoda: Brachyura) of India. Zootaxa.

[ref-83] Trivedi JN, Vachhrajani KD (2013a). Taxonomic account of genus *Scylla* (de Haan, 1833) from Gujarat State, India with two new records of species. Arthropods.

[ref-84] Trivedi JN, Vachhrajani KD (2013b). First record of two porcellanid crabs from Gujarat state, India (Crustacea: Decapoda: Porcellanidae). Journal of the Marine Biological Association of India.

[ref-85] Trivedi JN, Vachhrajani KD (2015). First record of brachyuran crab *Leptodius affinis* (De Haan, 1835) (Crustacea: Decapoda: Xanthidae) from the western coast of India. Marine Biodiversity Records.

[ref-86] Trivedi JN, Vachhrajani KD (2016a). On burrow morphology of the ghost crab, *Ocypode ceratophthalmus* (Decapoda: Brachyura: Ocypodidae) from sandy shore of Gujarat, India. International Journal of Marine Science.

[ref-87] Trivedi J, Vachhrajani K (2016b). On new record of *Pagurus kulkarni* Sankolli, 1962 (Crustacea: Anomura: Paguridae) from Gujarat, India. International Journal of Fisheries and Aquatic Studies.

[ref-88] Trivedi JN, Vachhrajani KD (2017). Seasonal burrow distribution of the ghost crab, *Ocypode ceratophthalmus* (Pallas, 1772), on Sandy Shores of Gujarat, India. International Journal of Marine Science.

[ref-89] Trivedi JN, Vachhrajani KD (2018). On new record of brachyuran crab *Scopimera crabicauda* Alcock, 1900 (Crustacea: Decapoda) from India. Journal of the Marine Biological Association of India.

[ref-90] ViñaTrillos N, Brante A, Urzúa Á (2023). Intraspecific variation in reproductive traits and embryo elemental composition of the crab *Hemigrapsus crenulatus* (Milne Edwards, 1837) across fluctuating coastal environments along Chilean coasts. Marine Environmental Research.

[ref-91] Wada S, Kitaoka H, Goshima S (2000). Reproduction of the hermit crab *Pagurus lanuginosus* and comparison of reproductive traits among sympatric species. Journal of Crustacean Biology.

[ref-92] Waiho K, Ikhwanuddin M, Abualreesh MH, Shu-Chien AC, Ishak SD, Jalilah M, Azmie G, Fazhan H (2021). Intra- and interspecific variation in sexual dimorphism patterns of mud crab genus *Scylla* along the equatorial region. Frontiers in Marine Science.

[ref-93] Wenner AM (1972). Sex ratio as a function of size in marine crustacea. The American Naturalist.

[ref-94] Zhang B, Yu C, Xu Y, Huang Z, Cai Y, Li Y (2023). Hepatopancreas immune response during different photoperiods in the Chinese mitten crab, *Eriocheir sinensis*. Fish & Shellfish Immunology.

[ref-95] Zimmerman TL, Felder DL (1991). Reproductive ecology of an intertidal brachyuran crab, *Sesarma* sp. (nr. *reticulatum*), from the Gulf of Mexico. The Biological Bulletin.

